# Alignment of Perceived Competencies and Perceived Job Tasks among Primary Care Managers

**DOI:** 10.3390/healthcare8010009

**Published:** 2019-12-27

**Authors:** Milica Dikic, Dejan Nikolic, Jovana Todorovic, Zorica Terzic-Supic, Milena Kostadinovic, Uros Babic, Marijana Gacevic, Milena Santric-Milicevic

**Affiliations:** 1 Center-School of Public Health and Health Management, Faculty of Medicine, University of Belgrade, 11000 Belgrade, Serbia; lokica82@yahoo.com (M.D.); jole6989@hotmail.com (J.T.); zoricaterzic37@gmail.com (Z.T.-S.); 2 Faculty of Medicine, University of Belgrade, 11000 Belgrade, Serbia; denikol27@gmail.com (D.N.); milena8250@hotmail.com (M.K.); milena8250@gmail.com (U.B.); marijana.marijana.g@gmail.com (M.G.); 3 University Children’s Hospital, 11000 Belgrade, Serbia; 4 Clinical Center of Serbia, 11000 Belgrade, Serbia

**Keywords:** management, competencies, planning and priority-setting, performance assessment, nurses, primary health care

## Abstract

In this study we aimed to explore how managers in primary health care (PHC) organizations assess their managerial knowledge and skills, as well as the importance of these competencies for their job, and to identify whether there is an alignment between these two perceptions; therefore, whether there is a need for management competency improvement. With this study, we tried to address a high demand for information about health managers, especially in health systems in low- and middle-income countries. In a sample of 58 primary health care centers (*n* = 106 managers) in Serbia, we used a basic managerial competency matrix consisting of the following six competencies—communication, team-building, planning and priority-setting, performance assessment, problem-solving, and leading. Managerial perception of the importance of their job tasks differs by educational level and managerial position. The best alignment between the importance of knowledge and skills was for communication and leading. The study pointed out that managers were aware of the necessity to improve their level of managerial competencies, particularly in the domains of planning and priority-setting, performance assessment, and problem solving. The study highlights the need for formal managerial education for managers in PHC settings and commitment to continuously evaluate and improve management competencies in order to better manage PHC.

## 1. Introduction

Primary health care (PHC) is a key component of a sustainable health care system [1,2,3]. Good management practice is necessary to improve the organization and provision of health care. Well-organized PHC is essential for the provision of health care that positively influences health of the population [2]. As PHCs have the responsibility for achievement of the universal health coverage as a part of the Sustainable Development Goals, it needs leadership capacity too [3,4]. The PHC should meet the health care needs of the members of the entire population over the course of their lives; therefore, skills like planning, priority setting, performance assessment, and problem solving are considered essential for managers at this level. Typically, management of PHC reflects the health policy regulations in a country, including the required level of ability for communicating and implementing the relevant evidence from research into practice. Worldwide, great efforts are made to strengthen management systems through assessment, education, and training for developing a comprehensive and integrated approach for quality of services provision in PHC [4,5,6,7,8,9,10,11,12,13].

Effective PHC leadership invests in building adequate teams of managers who would provide the appropriate competencies that are important for the successful functioning of healthcare facilities and the delivery of high-quality healthcare [4]. Management competencies are crucial and essential both on a strategic and operational level in a health care system [6,10]. On a strategic level, as creators of policies, managers supervise strategic directions, organize overall recourse allocation, and monitor health outcomes, while on an operational level they are responsible for finances, staff, equipment, supplies, and infrastructure [10]. Their primary responsibility is to plan, organize, lead, control, and develop recourses and partnerships, whether they are formally educated or not [5].

According to the World Health Organization (WHO), for an efficient and effective PHC, there is a need for an adequate number of managers with suitable competencies, the existence of a functional critical support system, and an optimal work environment [6]. Because primary health care reform is a worldwide imperative, WHO encourages countries to focus their health care systems towards strengthening the PHCs’ management. These efforts could improve the distribution of health care recourses along with reduction of their costs [6,7,8] and create PHC organizations as the most productive and rewarding working environments possible. Nowadays, healthcare organizations are in a dynamic environment that requires greater creativity and productivity, so the managers who lead them must have the competencies to plan and evaluate performance in order to deliver high quality healthcare in line with the rapid development of health technologies [14]. Over the past several years, there has been a growing focus on measuring competencies and performances in health care. Competencies are an integrated set of knowledge, skills, attitudes, and behaviors that one individual possesses, which enable effective performance of tasks and activities in certain occupations or functions in various complex situations [14,15,16,17,18,19,20,21]. Systematic measurement is important for the continuous progress of quality in health care, best management practice use, and management educations design [15]. The complexity of competences in health care is pointed out in several leadership models (the National Center for Healthcare Leadership model, the leadership model at the Cleveland Clinic [22], and the managerial matrix of core competencies developed by the Centers for Disease and Control in the United States (CDC-US) [20]), and leading change, transformation, continuous personal development, teamwork, and integrity are among numerous leadership attributes.

A new approach in measuring competencies is needed and should be oriented on how much competencies correspond to job requirements or how much influence they can have on performances of the facilities and quality of health care [19]. In the study of Monrad et al. [23], importance of competencies is stressed for both undergraduate and graduate medical education with the systematic and rigorous approach in their assessment. To support this, several groups worldwide developed programs identifying competencies that doctors should have, such as CanMEDs in Canada, that were adopted later in some European countries, for example Netherlands and Denmark [24,25], as well as The Outcome Project in the US, the Global minimum essential requirements in China [19], along with around 120 management training modules that were developed by WHO [15] and many others [14,15,16,17,18,20,21]. Although there are differences between these frameworks, managerial competencies are recognized as very important for success in the health care [14,15,16,17,18,19,20,21,24,25]. According to the CDC-US, a basic managerial competency matrix consists of the following six competencies—communication, team-building, planning and priority-setting, performance assessment, problem-solving, and leading [20].

By applying the CDC-US matrix of basic managerial competencies [20], in this study we aimed to explore how managers in PHC organizations assess their managerial knowledge and skills, as well as the importance of these competencies for their job, and identify whether there is an alignment between these two perceptions, and whether there is a need for management training. In this study, we tried to address a high demand for information about health managers, especially in health systems in low- and middle–income countries.

## 2. Materials and Methods

The research was conducted during the 2015 year in 58 PHC centers in Serbia. The study was approved by Master of Public Health Review Board of FMUB Centre-School of Public Health and Health Management (03/19 September 2016). These PHC centers represent 37% of the facilities of this kind in the country [26]. The criteria for inclusion in the study were that PHC centers had not previously organized management education and that managers in those PHC centers had not previously attended management courses. The participants were excluded from the analysis if they stated that they already had the managerial education. Participants were at managerial positions, such as directors, deputy directors, department heads, head nurses, and lower managerial positions, such as, heads of administrative or technical services, and IT units. The final analysis included 106 respondents (106/116, the response rate of 91.4%). The research instrument was adopted from the CDC-US structured questionnaire and translated into Serbian and culturally adjusted [15,20]. It consists of 36 questions divided into two parts (Supplementary Material in Serbian language-S1). The first part contains questions about socio-demographic characteristics and a piece of basic information about the participants’ education and work position, while the second part refers to knowledge and skills for their job from six core domains of competencies—communication, team-building, planning and priority-setting, performance assessment, problem-solving, and leading (Table 1).

Respondents were asked to self-assess the level of importance (‘importance’) of knowledge and skills for their job as well as their level knowledge and skills to perform the job (‘knowledge and skills’) on a five-point Likert scale, where 1 represents the participant perception that knowledge and skills are not important for their job/participant has no particular knowledge and skill and 5 represents that a participant perceives knowledge and skills as very important/a participant perceives he/she has very high knowledge and skills. The variable ‘importance’ was presented as an average score on the Likert scale for each of the six core domains stated under the importance of knowledge and skills for the job. The variable ‘knowledge and skills’ was presented as an average score for each of the six core domains stated under the possessed knowledge and skills for the job. Alignment of perceived importance and perceived knowledge and skills for each of the six core domains of job competencies was estimated by the formula [15,20]:

A= I^2^ – KS^2^

where A represents the difference level, I represents the perceived importance for the job, and *KS* represents the perceived knowledge and skills. The higher the difference *A* the lower the alignment between the *I* and the *KS*.

In total, 12 variables were analyzed. These were age, gender, length of work experience, length of experience on a managerial position, current managerial position, level of education (high school, college, faculty, and postgraduate level), and the six main domains of competencies (communication, team-building, planning and priority setting, performance assessment, problem-solving, and leading). Participants were divided into two groups based on age (35 years or younger and older than 35 years). According to the length of work experience, the participants were divided into five groups (the first group was with 0–5 years of work experience, the second with 6–10 years, the third with 11–15 years, the fourth with 16–20 years, and in the fifth were managers with more than 20 years of work experience). Based on the length of experience on the managerial position, the participants were divided into two groups (the first group of managers with less than one year of experience and the second group of managers with more than one year of experience).

The data were analyzed by methods of descriptive (absolute and relative values, measures of central tendency, variability, and Cronbach’s Alpha) and analytical statistics. The Cronbach’s Alpha coefficient confirmed the reliability of the consistency of the instrument scales: the lowest Cronbach’s Alpha was 0.72 for domain leading and the greatest was 0.91 for domain planning and priority-setting (Table 1). Competency rating differences between participants were analyzed with Kruskal–Wallis and Mann Whitney U test. The results were considered statistically significant at the level of *p*-value 0.05 or less. Statistical analysis was performed with SPSS Statistics 21.0 (IBM Corporation, Armonk, NY, USA).

## 3. Results

### 3.1. Study Population Characteristics

All 106 managers completed the questionnaire. The oldest participant was 62 years old, while the youngest was 29. The average age was 47.84 years (SD 8.57). The majority of the study population were females (n = 72 (67.9%)). The participants were categorized by the managerial function they perform. The majority of participants were directors (32.1%), followed by assistant managers (22.6%), deputy directors (16.0%), heads of department (15.1%), and head nurses (11.3%). Fifty-four participants (50.9%) had at least one year of postgraduate studies, 37 (34.9%) had faculty education, 12 (11.3%) had college education, and only 3 (2.8%) participants had high school education. The largest number of managers had 20 years of working experience and more (39.6%) and was older than 40 years (36.8%), while 54.8% perform a managerial function for longer than one year.

### 3.2. Core Domains of Managerial Competencies of PHC Managers in the Study

The perceived level of importance of knowledge and skills for the job and self-perceived knowledge and skills of managers in the study are presented in Table 2. Average scores for all core domains of managerial competencies were higher than 3 (i.e., moderate level of competency importance for the job and moderate level of knowledge and skills for the job). Participants perceived that knowledge and skills for team-building have the highest level of importance for their job (I = 4.79 ± 0.45) and they also self-perceived their knowledge and skills for team building as the highest (KS = 4.31 ± 0.61).

The best alignment between the perceived level of importance and the perceived level of knowledge and skills was assessed for communication and leading (A = 3.04; and A = 3.93, respectively), while the worst alignment was identified between the perceived level of importance and the perceived level of knowledge and skills for planning and priority setting (A = 5.21).

### 3.3. Core Domains of Managerial Competencies According to the Socio-Demographic Characteristics of PHC Managers in the Study

The socio-demographic characteristics of PHC managers in the study and the alignment of the perceived level of importance and the possessed knowledge and skills for the job are presented in Table 3. Level of education and managerial position are important characteristics for aligning the perceived level of importance and perceived knowledge and skills regarding some job competencies. Manager’s level of education makes a significant difference for leading competency (*p* < 0.028), i.e., postgraduate educated managers have a greater alignment of the perceived importance of the leading for the job and the perceived level of leading knowledge and skill than the managers with other levels of education. In addition, the managerial position plays a significant role in the communication domain (*p* < 0.048), i.e., among all managers, the head nurses have the least alignment of the perceived importance of the communication for the job and the perceived level of knowledge and skills for communication (Table 3).

## 4. Discussion

In this study, we explored how managers in PHC organizations assess their managerial knowledge and skills, as well as the importance of these competencies for their job. We were also interested in understanding whether there is an alignment between these two perceptions, or whether there is a need for management competency improvement. The alignment of competencies with the managerial job is needed for informed decision-making and efficient resource allocation in PHC, in order to help them organize and deliver quality and optimal health services for the population [10,27,28].

The managers perceive team building as the most important competency for successful PHC and communication as the least important. On average, health managers self-assessed their level of competency as moderate but still below the level of importance of the job they perform. The best alignment between the importance and knowledge and skills was for communication and leading. The perceived importance of knowledge and skills for the job was the highest for team-building and problem solving, while the highest self-perceived skills and knowledge were for the team-building and leading. Our study showed that the smallest alignment between the perceived importance of knowledge and skills and self-perceived knowledge and skills among managers in Serbia was in planning and priority setting, followed by assessing performance and team-building. The previous study about competencies of managers in PHCs of Belgrade, the capital of Serbia, had similar results [15], indicating the insufficient education in the field of management. Doctors and nurses included in the management of PHC centre also need competency building to be able to well organize a business environment and ensure the competitive advantage of PHC services at the current market [6,15,17]. Managers’ perception of the importance of their job tasks differs by educational level and managerial position. In our study, there was a statistically significant difference in the alignment for the perceived importance of leading for a job and self-perceived knowledge and skills possessed for leading according to the educational level. The minor alignment was identified among managers with a college education, while the largest alignment was among managers with postgraduate studies. The managers with postgraduate studies might be regarded as highly competent and perceived as experts in the field, which might then lead to enhancement of the prestige of their institution, resource allocation, and cooperation [29].

According to managerial position, the smallest alignment in perceived importance of skills and knowledge and self-perceived skills and knowledge was among head nurses. The minor alignment was among head nurses regarding communication competence, indicating the need for formal education to develop proactive knowledge and essential skills that will enhance professional nursing practice and nursing relationships with various stakeholders [30]. Researchers worldwide emphasize the importance of education and training as tools for improvement of core managerial competencies [15]. A study of nursing manager competencies from South Africa determined that even if the nursing managers have the experience, there is still a need for their continued professional education [16]. This type of education with specific aims could improve competencies, reduce the competency gaps and improve the decision-making process by upgrading skills in finding and using adequate information or research evidence [15,28].

Another very important study finding is that Serbian managers well perceived the necessity to improve their level of managerial competencies. In a recent study, a nonconformity of perceptions on the same issues was found between managers on different positions, namely, although most of the nursing managers consider themselves competent from all domains of competencies, their superiors and subordinates evaluate their competencies lower and think they need education [16]. On the other hand, the lack of adequate managerial training can also lead to the lower self-perceived competencies [15]. The Serbian managers need competency building in particular in the domains of planning and priority-setting, performance assessment, and problem solving, since there is a large difference between the importance of these competences for job and the self-perceived level of knowledge and skills. The lack of quality data and support systems for information processing and systematic assessment and insufficient top management buy-in [31,32,33] may also contribute to these differences. Managers in our study also had low competencies for team-building, and this deficiency, according to other authors [34], might contribute to an increase in health care costs.

The studies from China and Thailand resulted in the formation of competency frameworks where competencies are systematically and specifically developed and measurable [19,35]. Not so long ago, in 2013, the study conducted in Canada, Australia, Netherlands, and Denmark pointed out that introducing managerial education and training in formal education should be mandatory in accordance with the needs of health professionals [24]. The recent research of managerial competencies from Belgrade pointed out that managerial education along with regulations, policies, and procedures can remarkably upgrade managerial competencies and reduce the gap between perceived importance of skills and knowledge and competency level, which can result in resource optimization in health care services, which can contribute to better health outcomes [15]. Nonetheless, learning by working is a predominant method of gaining managerial competencies among doctors and nurses in Serbia, despite a spectrum of formal management training offered in the country. As of 2022, the top managers of health care institutions in the public sector will be required to obtain formal management education according to the new regulations in the health sector. Therefore, it is important that managers are able to identify necessary job competencies [17,24,35,36] and to upgrade professional behavior and values, communication, team-work, managing, and technical procedural skills, including planning, priority setting, assessing performance, and problem solving. This stresses the importance of aligning management education and the needs of health care organizations among health professionals in managerial positions in order to achieve a state in which most managers have already necessary education in the field of management. The study emphasizes that health care managers need formal management education as e.g., health workers, architects, and lawyers need the qualifications to be able to work in practice.

There are several limitations to this study. PHC centers included in this study represent 37% of the total PHC centers in Serbia [26], so conclusions should not be generalized for PHC managers in the whole country. In the lack of formally requested managerial competencies in Serbia, we chose the CDC-US [20] questionnaire to measure the alignment of job tasks with competencies according to the model of managerial competencies from public health institutions and also in accordance with priorities for the development of PHC competencies that were formulated by the Ministry of Health of Republic Serbia [37]. Use of the Likert scale in the questionnaire could influence the results in the sense of letting respondents choose an option that suits their perception at the moment. Self-assessment can be often overestimated or underestimated as well, which could influence the findings [15,16].

The significance of this research is that it is contributing to existing worldwide efforts for improving the managerial competencies of health professionals. It also addresses an interest in methods for assessing the managerial potential in PHC. Most studies refer to assessing clinical competencies of health professionals; however, doctors, nurses, and other health professionals also have a great role in health care management [5,31]. This study points out the way to further manage competency improvement of health care managers. A successful manager achieves important results for the organization by encouraging and leading the performance assessment. Therefore, regular monitoring and periodic assessment of the performance, creating a culture of the performance assessment, could help the managers understand if they were on the right way to improving their professionalism [38] and PHC outcomes [39].

Managerial competencies of all profiles of health care professionals should be a priority for future research to improve the provision of health services in PHC. Research evidence from this study could be useful for policy makers for the formulation of adequate frameworks for managerial performance assessment and for the identification of the needs for continued managerial education in order to provide competent PHC managers. Human resources development leaders should advocate investments in managerial competencies and should give much higher priority to management development [3,4,5,38,40]. Investment in the development of professional staff of quality managers and managerial teams is a key point for goal realization in PHC in Serbia, as well as in many other countries that are to meet the majority of an individual’s health needs over the course of their life and leave no one in their community behind [3].

## 5. Conclusions

On average, health managers in Serbia self-assessed their level of competency as moderate but still below the level of importance of the job they perform. The perceived importance of competencies for the job was the highest for team-building and problem solving, but there was the smallest alignment between the perceived importance and self-perceived knowledge and skills in planning and priority setting, followed by assessing performance. The results of this research can have an impact on the development of management in the PHC of Serbia as well as in the world, in terms of providing new evidence on the need to increase managers’ awareness of their own competencies and the obligation to improve them in order to better manage the PHC. The study emphasizes that healthcare managers need formal management training and qualifications to be able to work in practice.

## Figures and Tables

**Table 1:** Questionnaire items.

Part I Personal Data (Age and Gender, Level of Education, Work Experience (Years), Managerial Position and Managerial Experience (Years), and Education in Management)		
Part II Perception at a Five-Point Likert Scale of the Importance and Knowledge and Skills (i.e., 1-not Important/no Knowledge and Skills to 5-Very Important/Very High Knowledge and Skills)		
Competency	Cronbach’s Alpha coefficient	
	The self-perceived level of the importance of the knowledge and skills for the job (I = ‘importance’)	The self-perceived level of the knowledge and skills for the job (KS = ‘knowledge and skills’)
Communication: Oral presentation, Interview or writing articles to media, Planning and implementing communication programs, and Designing a long-term communication strategy	0.742	0.861
Team-building: Being a team member; Leading, building and managing teams	0.616	0.877
Planning and priority setting: Setting priorities; Establishing work plans; Applying decision analysis techniques; Designing programs, and strategic, business, and work plans	0.793	0.883
Assessing performance: Assessing and evaluating performance of employees, of the program, and budget expenditures	0.778	0.797
Problem-solving: Identifying problems; Analyzing root causes and factors; Designing corrections, and Preventing and solving problems with employees and team	0.648	0.813
Leading: Aligning in teamwork; Motivating employees; and Sharing the vision and mission	0.792	0.827
Other competencies	−	−

**Table 2:** Perceived level of importance of knowledge and skills for the task and self-perceived knowledge and skills of managers in primary health care centers.

Core Domains of the Knowledge and Skills	Importance (I) X ± SD (95% CI)	Knowledge and Skills (KS) X ± SD (95% CI)	The Difference (A = I^2^-KS^2^) X ± SD (95% CI)
Communications	4.40 ± 0.71 (4.24–4.55)	4.03 ± 0.76 (3.86–4.20)	3.04 ± 5.80 (1.76–4.31)
Team building	4.79 ± 0.45 (4.69–4.89)	4.31 ± 0.61 (4.18–4.44)	4.24 ± 5.49 (3.03–5.45)
Planning and priority setting	4.65 ± 0.54 (4.53–4.77)	4.03 ± 0.66 (3.88–4.18)	5.21 ± 5.27 (4.05–6.37)
Assessing performance	4.54 ± 0.57 (4.42–4.66)	4.00 ± 0.65 (3.86–4.14)	4.52 ± 5.35 (3.35–5.70)
Problem solving	4.68 ± 0.46 (4.58–4.78)	4.20 ± 0.61 (4.06–4.33)	4.13 ± 5.31 (2.96–5.30)
Leading	4.69 ± 0.41 (4.60–4.78)	4.22 ± 0.61 (4.09–4.36)	3.93 ± 4.86 (2.87–5.00)

**Table 3:** Differences in the perceived importance of knowledge and skills and self-perceived knowledge and skills by sociodemographic characteristics of managers in studied primary health care (PHC).

Managers’ Sociodemographic Characteristics	Core Competencies Domains (A = I^2^-KS^2^) X ± SD (95% CI)					
	Communication	Team Building	Planning and Priority Setting	Performance Assessment	Problem Solving	Leading
Gender						
Male	3.22 ± 7.43 (−0.16–6.60)	4.02 ± 5.85 (1.36–6.68)	5.58 ± 6.46 (2.64–8.52)	4.44 ± 6.10 (1.67–7.22)	4.62 ± 5.72 (2.02–7.22)	4.41 ± 5.23 (2.02–6.79)
Female	4.16 ± 5.12 (2.63–5.7)	5.09 ± 5.59 (3.41–6.80)	6.07 ± 4.78 (4.63–7.50)	4.83 ± 5.04 (3.32–6.35)	4.89 ± 5.28 (3.30–6.47)	4.06 ± 4.72 (2.64–5.48)
Age						
<35 years	3.74 ± 10.63 (−6.09–13.57)	3.78 ± 6.40 (−2.15–9.70)	6.42 ± 7.88 (−0.87–13.70)	2.79 ± 4.97 (−1.80–7.38)	4.86 ± 4.04 (1.12–8.59)	4.11 ± 3.17 (1.18–7.04)
≥35 years	2.97 ± 5.26 (1.76–4.18)	4.29 ± 5.45 (3.03–5.54)	5.10 ± 5.02 (3.94–6.25)	4.68 ± 5.39 (3.44–5.92)	4.06 ± 5.43 (2.81–5.31)	3.92 ± 5.00 (2.76–5.07)
Education						
High school	2.96 ± 4.56 (−8.44–14.36)	1.29 ± 4.42 (−9.69–12.28)	7.20 ± 2.54 (−15.61–30.02)	3.85 ± 4.64 (−7.67–15.37)	3.93 ± 6.21 (−11.50–19.35)	4.50 ± 6.36 * (−52.68–61.68)
College	5.32 ± 9.24 (−0.55–11.19)	5.92 ± 6.43 (1.83–10.00)	7.14 ± 5.76 (3.27–11.01)	5.68 ± 5.05 (2.47–8.89)	6.18 ± 4.80 (3.13–9.22)	6.92 ± 4.07 * (4.33–9.50)
Faculty	1.61 ± 5.75 (−0.33–3.56)	2.46 ± 6.02 (0.32–4.59)	4.84 ± 5.55 (2.87–6.81)	4.49 ± 5.60 (2.59–6.39)	4.03 ± 5.37 (2.21–5.85)	4.62 ± 4.46 * (3.06–6.18)
Post graduate studies	2.10 ± 5.20 (0.64–3.56)	3.52 ± 5.46 (1.99–5.04)	4.32 ± 4.93 (2.89–5.75)	4.41 ± 5.81 (2.79–6.03)	4.14 ± 5.86 (2.51–5.77)	2.75 ± 5.22 * (1.26–4.23)
Years of work experience						
0–5	4.87 ± 4.15 (−32.45–42.20)	10.28 ± 1.81 (−5.96–26.51)	8.72 ± 1.95 (−8.78–26.23)	5.33 ± 7.54 (−62.43–73.10)	5.89 ± 4.40 (−33.64–45.42)	7.33 ± 0.47 (3.09–11.57)
6–10	3.18 ± 9.32 (−6.60–12.96)	4.04 ± 9.52 (−5.95–14.02)	5.72 ± 7.86 (−2.53–13.98)	6.68 ± 5.35 (1.07–12.30)	8.43 ± 5.52 (2.63–14.22)	3.70 ± 3.46 (0.07–7.34)
11–15	5.49 ± 7.11 (1.39–9.60)	7.02 ± 5.78 (3.68–10.36)	8.99 ± 5.96 (5.55–12.44)	5.18 ± 4.56 (2.55–7.81)	7.95 ± 5.86 (4.57–11.34)	7.13 ± 6.12 (3.59–10.66)
16–20	2.78 ± 5.93 (−0.80–6.37)	3.72 ± 5.25 (0.55–6.89)	4.36 ± 5.14 (1.25–7.46)	3.29 ± 5.69 (−0.15–6.73)	3.95 ± 4.72 (1.10–6.80)	2.85 ± 4.63 (0.05–5.65)
>20	3.64 ± 4.7 (1.90–5.39)	3.93 ± 4.77 (2.18–5.68)	5.03 ± 4.28 (3.46–6.59)	4.67 ± 5.63 (2.60–6.73)	2.96 ± 4.69 (1.24–4.70)	3.27 ± 4.22 (1.73–4.82)
Years of managerial experience						
<1 year	5.25 ± 6.77 (1.98–8.51)	4.58 ± 5.15 (2.09–7.06)	6.33 ± 6.33 (3.28–9.38)	3.60 ± 5.45 (0.97–6.23)	4.55 ± 6.29 (1.52–7.58)	3.07 ± 4.79 (0.76–5.38)
≥1 year	3.30 ± 5.50 (1.68–4.91)	4.82 ± 5.89 (3.08–6.55)	5.74 ± 4.94 (4.29–7.19)	5.16 ± 5.31 (3.60–6.71)	4.90 ± 5.04 (3.42–6.38)	4.61 ± 4.86 (3.18–6.04)
Managerial position						
Director	3.60 ± 5.03 * (1.37–5.83)	5.44 ± 6.24 (2.67–8.21)	5.67 ± 4.47 (3.69–7.66)	4.45 ± 4.88 (2.28–6.62)	4.56 ± 6.33 (1.75–7.37)	3.48 ± 6.02 (1.30–5.65)
Assistant director	0.84 ± 6.42 * (−4.52–6.21)	3.98 ± 4.76 (0.00–7.97)	3.64 ± 6.86 (−2.10–9.37)	2.22 ± 7.29 (−3.87–8.32)	2.80 ± 6.36 (−2.51–8.12)	2.88 ± 5.01 (0.10–5.66)
Deputy director	1.11 ± 5.78 * (−1.96–4.19)	3.29 ± 6.72 (−0.29–6.87)	4.70 ± 5.63 (1.70–7.70)	4.68 ± 5.73 (1.63–7.73)	4.10 ± 5.39 (1.71–6.50)	3.99 ± 4.40 (1.65–6.34)
Head of the department	6.22 ± 4.37 * (3.28–9.16)	5.76 ± 4.81 (2.52–8.99)	7.89 ± 4.68 (4.74–11.03)	5.89 ± 4.59 (2.81–8.97)	5.69 ± 4.06 (2.96–8.42)	4.37 ± 4.36 (2.05–6.69)
Head nurse	9.18 ± 5.54 * (4.92–13.44)	5.07 ± 3.99 (2.01–8.14)	8.25 ± 5.52 (4.01–12.50)	6.16 ± 5.03 (2.29–10.03)	7.28 ± 3.62 (4.50–10.07)	6.40 ± 3.56 (4.01–8.79)

* *p* < 0.05-Kruskal–Wallis test.

## Supplementary Materials

The following are available online at https://www.mdpi.com/2227-9032/8/1/9/s1. Table S1: Serbian version of questionnaire.

## References

[1] World Health Organization Declaration of Alma Ata. Proceedings of the International Conference on Primary Health Care.

[2] Starfield B., Shi L., Macinko J. (2005). Contribution of Primary Care to Health Systems and Health. Milbank Q..

[3] World Health Organization and the United Nations Children’s Fund (2018). Declaration The Global Conference on Primary Health Care, Astana, Kazakhstan. https://www.who.int/docs/default-source/primary-health/declaration/gcphc-declaration.pdf.

[4] Dussault G., Kawar R., Castro L.S., Campbell J. (2018). Building the Primary Health Care Workforce of the 21st Century—Background Paper to the Global Conference on Primary Health Care: From Alma-Ata Towards Universal Health Coverage and the Sustainable Development Goals.

[5] The World Health Organization (2005). Strengthening Management in Low-Income Countries. The “Making Health Systems Work”.

[6] The World Health Organization (2007). Towards Better Leadership and Management in Health: Report on an International Consultation on Strengthening Leadership and Management in Low-Income Countries. The “Making Health Systems Work”.

[7] The World Health Organization (2008). The World Health Report 2008: Primary Health Care Now More Than Ever.

[8] Starfield B. (2009). Toward international primary care reform. CMAJ.

[9] Pan American Health Organization (2009). Primary Health Care—Health Based Systems: Strategies for the Development of Primary Health Care Team.

[10] The World Health Organization (2009). Who Are Health Managers. Case Studies from Three African Countries.

[11] Stigler F.L., Starfield B., Sprenger M., Salzer H.J., Campbell S.M. (2013). Assessing primary care in Austria: Room for improvement. Fam. Pract..

[12] Ellner A.L., Phillips R.S. (2017). The Coming Primary Care Revolution. J. Gen. Intern. Med..

[13] Tabrizi J.S., Gharibi F. (2019). Primary health care accreditation standards: A systematic review. Int. J. Health Care Qual. Assur..

[14] Stefl M.E. (2008). Common Competencies for All Healthcare Managers: The Healthcare Leadership Alliance Model. J. Healthc. Manag..

[15] Santric Milicevic M.M., Bjegovic-Mikanovic V.M., Terzic-Supic Z.J., Vasic V. (2009). Competencies gap of management teams in primary health care. Eur. J. Public Health.

[16] Munyewende P.O., Levin J., Rispel L.C. (2016). An evaluation of the competencies of primary health care clinic nursing managers in two South African provinces. Glob. Health Action.

[17] Lane D.S., Ross V. (1998). Defining competencies and performance indicators for physicians in medical management. Am. J. Prev. Med..

[18] Capital Health Primary Health Care (2012). Primary Health Care Competency Framework.

[19] Zhao L., Sun T., Sun B.Z., Zhao Y.H., Norcini J., Chen L. (2015). Identifying the competencies of doctors in China. BMC. Med. Educ..

[20] The Sustainable Management Development Program, Core Management Competencies for Public Health Managers, Atlanta US: Coordinating Office for Global Health, The Centers for Disease Control and Prevention. https://stacks.cdc.gov/view/cdc/13701/cdc_13701_DS1.pdf?.

[21] ACHE Healthcare Executive Competencies Assessment Tool (2019). Healthcare Leadership Alliance and the American College of Healthcare Executive. https://www.ache.org/-/media/ache/career-resource-center/competencies_booklet.pdf.

[22] Stoller J.K. (2018). Developing Physician Leaders: A Perspective on Rationale, Current Experience, and Needs. Chest.

[23] Monrad S.U., Mangrulkar R.S., Woolliscroft J.O., Daniel M.M., Hartley S.E., Gay T.L., Highet A., Vijayakumar N., Santen S.A. (2019). Competency Committees in Undergraduate Medical Education: Approaching Tensions Using a Polarity Management Framework. Acad. Med..

[24] Berkenbosch L., Schoenmaker S.G., Ahern S., Sojnaes C., Snell L., Scherpbier A.J., Busari J.O. (2013). Medical residents’ perceptions of their competencies and training needs in health care management: An internal comparison. BMC. Med. Educ..

[25] Frank J.R. (2005). The CanMEDS 2005 Physician Competency Framework. Better Standards. Better Physicians. Better Care.

[26] Institute of Public Health of Serbia (2016). Health Statistical Yearbook of Republic of Serbia 2015.

[27] Ellen M.E., Leon G., Bouchard G., Lavis J.N., Ouimet M., Grimshaw J.M. (2013). What supports do health system organizations have in place to facilitate evidence—Informed decision—Making? A qualitative study. Implement. Sci..

[28] Hogg W., Dyke E. (2011). Improving measurement of primary care system performance. Can. Fam. Physician.

[29] Anderson P., Pulich M. (2002). Managerial competencies necessary in today’s dynamic health care environment. Health Care Manag. (Frederick).

[30] Nokuthula S.M., Ulutasdemir N. (2018). Effective Communication in Nursing. Nursing.

[31] Thokala P., Devlin N., Marsh K., Baltussen R., Boysen M., Kalo Z., Longrenn T., Mussen F., Peacock S., Watkins J. (2016). Multiple Criteria Decision Analysis for Health Care Decision Making—An Introduction: Report 1 of the ISPOR MCDA Emerging Good Practices Task Force. Value Health.

[32] Kapiriri L. (2018). Stakeholder involvement in health research priority setting in low income countries: The case of Zambia. Res. Involv. Engagem..

[33] Hipgrave D.B., Laksmono L.H., Koemarasakti G.M., Nandy R., Setiawan B., Hermawan L., Marbun D. (2018). District team problem solving as an approach to district health programme planning: A review, and survey of its status in selected districts in Indonesia. Health Policy Plan.

[34] Miller C.J., Kim B., Silverman A., Bauer M.S. (2018). A systematic review of team-building interventions in non-acute healthcare settings. BMC Health Serv. Res..

[35] Kitreerawutiwong K., Sriruecha C., Laohasiriwong W. (2015). Development of competency scale for primary care managers in Thailand: Scale development. BMC. Fam. Pract..

[36] Slipicevic O., Masic I. (2012). Management Knowledge and Skills Required in the Health Care System of Federation Bosnia and Herzegovina. Mater. Sociomed..

[37] Ministry of Health of the Republic of Serbia (2009). Better Primary Health Care for Us; Policy Directions to Strengthen the Primary Health Care System in Serbia, from 2010 to 2015.

[38] Curzi Y., Fabbri T., Scapolan A.C., Boscolo S. (2019). Performance Appraisal and Innovative Behavior in the Digital Era. Front. Psychol..

[39] Espinosa-González A.B., Delaney B.C., Marti J., Darzi A. (2019). The impact of governance in primary health care delivery: A systems thinking approach with a European panel. Health Res. Policy Sys..

[40] European Commission Directorate General for Health and Food Safety (2018). A New Drive for Primary Care in Europe: Rethinking the Assessment Tools and Methodologies. Report of the Expert Group on Health Systems Performance Assessment. Europe Union. https://primaerversorgung.org/wp-content/uploads/2017/04/2018_Health-Systems-in-the-EU-Commission-publishes-report-on-Primary-Care.pdf.

