# Supplementary material for: Alignment of Perceived Competencies and Perceived Job Tasks among Primary Care Managers
Source: Healthcare (Basel). 2019 Dec 27;8(1):9. doi: 10.3390/healthcare8010009 (PMC7151202; doi:10.3390/healthcare8010009)
Supplement: Supplementary file 1 [file healthcare-08-00009-s001.pdf]

Supplementary material in Serbian language S1.

Medicinski fakultet Univerziteta u Beogradu
Institut za socijalnu medicinu

PROGRAM UNAPREĐENJA MENADŽMENTA ZDRAVSTVENIM USTANOVAMA U BEOGRADU

UPITNIK ZA UČESNIKE PROGRAMA

I

Godine života:	Pol: m ž
1. Ustanova:	Godine radnog staža:
2. Vaše radno mesto (molimo, navedite i rukovodeću funkciju):	
3. Koliko ste dugo na tom radnom mestu:	
4. Molimo, navedite stepene obrazovanja koje ste stekli:	
5. Molimo, navedite evenutualnu obuku iz menadžmeta koju ste prethodno pohađali	

Tema	broj dana	kada

II Molimo da za svaki od 6 tipova zadataka radnog mesta u koloni A, zakružite odgovore na pitanja u kolonama B i C.

KOLONA A	KOLONA B					KOLONA C				
**Zadaci Vašeg radnog mesta**	**Važnost zadatka za uspešan rad**					**Stepen veštine koji posedujete za obavljanje zadatka**				
	**Nije Važan**<br>**1**	**Retko važan**<br>**2**	**Ponekad važan**<br>**3**	**Često Važan**<br>**4**	**Vrlo Važan**<br>**5**	**Nemam Veštinu**<br>**1**	**Nizak**<br>**2**	**Srednji**<br>**3**	**Viši**<br>**4**	**Visok**<br>**5**
**1. KOMUNIKACIJE**										
Usmena prezentacija pred 8 Ili više osoba										
Bilti intervjusian ili pisati članak za medije (TV, radio, novine)										
Planirati i primeniti program komunikacije u organizaciji ili sa njenim korisnicima.										
Dizajnirati dugoročnu komunikacionu strategiju programa ili organizacione jedinice										
**2. GRAĐENJE TIMA**										
Biti član tima i radom doprinositi timu										
Voditi tim odgovoran za ključne organizacione aktivnosti										
Graditi timove i rukovoditi timovima zaduženim da proizvedu ključne organizacione rezultate										
**3. PLANIRANJE I USPOSTAVLJANJE PRIORITETA**										
Postaviti prioritete u individualnom radu, razviti lični plan rada, i rukovoditi ličnim vremenom efektivno										
Postaviti prioritete i radne planove za druge										
Primeniti tehnike analize odlučivanja da bi postavili prioritete programa i/ili radne jedinice										
Napraviti detaljan plan rada službe sa raspodelom zadataka, rasporedom i alokacijom resursa										
Uraditi strateški plan										
Planirati potrebe za kadrovima za period od 3 ili više										

godina		
Uraditi plan poslovnog slučaja		

**Zadaci Vašeg radnog mesta**	**Važnost zadatka za uspešan rad**					**Stepen veštine koji posedujete za obavljanje zadatka**				
	**Nije Važan**<br><br>**1**	**Retko važan**<br><br>**2**	**Ponekad važan**<br><br>**3**	**Često Važan**<br><br>**4**	**Vrlo Važan**<br><br>**5**	**Nemam Veštinu**<br><br>**1**	**Nizak**<br><br>**2**	**Srednji**<br><br>**3**	**Viši**<br><br>**4**	**Visok**<br><br>**5**
**4. PROCENA PERFORMANSI/ IZVRŠENJA/UČINKA**										
Procenjivati performanse zaposlenih pod vašom supervizijom, pružiti korisnu povratnu informaciju i obuku										
Evaluirati i procenjivati performanse programa ili delova programa										
Evaluirati i procenjivati performanse programa ili delova programa u odnosu na troškove budžeta										
**5. REŠAVANJE PROBLEMA**										
Rešavati probleme ponašanja i rada zaposlenih i tima										
Identifikovati probleme u toku odvijanja programa ili delova programa, analizirati osnovne uzroke, i dizajnirati odgovor da bi rešili program										
Utvrditi i analizirati faktore u spoljnjem okruženju koji mogu uticati na program i/ili preduzeti mere za njihovu korekciju i prevenirati probleme.										
**6. VOĐENJE**										
Motivisati zaposlene na individualne akcije										
Razgovarati o viziji i misiji sa timovima i radnim grupama										

Povezati i motivisati radne timove zajedničkom svrhom		
**7. DRUGO?**		
